# Relationship between Anger Expression in Mothers and Acceptance of Behavior Management Techniques in Pediatric Dentistry

**DOI:** 10.30476/dentjods.2022.92626.1665

**Published:** 2023-03

**Authors:** Reihaneh Faghihian, Mehdi Jafarzadeh, Mona Esmaeili, Narges Malek Mohamamdi, Mohammad Hossein Nikbakht

**Affiliations:** 1 Dept. of Pediatric Dentistry, Dental Research Center, Dental Research Institute, Isfahan University of Medical sciences, Isfahan, Iran; 2 Postgraduate Student Dept. of Pediatric Dentistry, Dental Research Center, School of Dentistry, Isfahan University of Medical Sciences, Isfahan, Iran; 3 Dentistry Student, Student Research Committee, School of Dentistry, Isfahan University of Medical Sciences, Isfahan, Iran

**Keywords:** Anger expression, Behavior management technique, Child, Parental acceptance, Pediatric dentistry

## Abstract

**Statement of the Problem:**

The parental acceptance rate of the behavior management methods used in pediatric dentistry is influenced by various factors.

**Purpose:**

This study was aimed to investigate the relationship between mothers’ anger expression and their acceptance of behavior management methods.

**Materials and Method:**

In this cross-sectional study, 110 mothers of children who had less than 12 years of age were recruited. They completed Spielberg’s state-trait anger expression inventory (STAXI). The respondents watched an educational video that included explanations of seven behavior management methods. Then, they reported their acceptance of each of these methods using the visual analog scale (VAS).

**Results:**

The acceptance rates of behavior management methods from the highest to lowest included tell-show-do, pre-appointment behavior shaping, mouthpiece, voice control, general anesthesia, active restraint, and passive restraint, respectively. There was only a significant inverse relationship between the acceptance of the tell-show-do method and the anger expression (*p*< 0.05). There was no significant relationship between the acceptance of behavioral management methods and the previous history of a pediatric dental visit, age, and mothers’ education.

**Conclusion:**

There was a significant inverse relationship between acceptance of the tell-show-do behavioral management method and mothers’ anger expression. However, there is no significant relationship between other methods and mothers’ anger expression. According to this study, there was a relation between mothers' ages and their acceptance of using a mouthpiece.

## Introduction

Dental fear is associated with many problems in children's behavior during and even after dental treatment [ 1 - 2 ]. Reducing dental fear, behavior management techniques are the basis for proper communication between the dental team and the young patients [ 2 - 3 ]. Pre-appointment behavior shaping, tell-show-do, voice control, mouthpiece, active restraint, passive restraint, and general anesthesia are common behavior management techniques [ 4 ]. Given the role of parents in the treatment triangle in pediatric dentistry and also the need for informed parental consent, the therapies and behavior management techniques should be accepted by the child's parents [ 5 - 7 ]. It seems that acceptance of different behavior management methods has changed over time, and education has gained a more important role in the growing acceptance of methods such as tell-show-do and general anesthesia [ 8 ]. As parents’ behaviors, practices, and desires, and parenting styles continue to change and adapt, behavior management practices in pediatric dentistry need to improve as well [ 6 ].

Anger expression and control, as a personality trait in parents, along with its effects can play a pivotal in behavior management in pediatric dentistry. Anger is defined as a set of relatively specific physiological emotions, cognitions, and reactions that are associated with a desire to harm a goal [ 9 ]. Anger can affect people's views, beliefs, thoughts, logic, choices, judgments, and decisions. Therefore, it can change people’s acceptance of a specific subject [ 9 ]. It seems that the occurrence and management of anger in parents affect the acceptance of behavior management methods in dentistry. Hence, observation and correct interpretation of anger experience, expression, and control in mothers can help to gain a better understanding of their perspective toward and acceptance of behavior management methods in dentistry. This study was aimed to investigate the relationship between mothers’ anger expression and their acceptance of behavior management methods.

## Materials and Method

### Ethics

The Ethics Committee of Isfahan University of Medical Sciences has been approved this study (IR.MUI.RESE-ARCH.REC.1399.409). 

### Participants

In this cross-sectional study, 110 mothers of children fewer than 12 years of age, who were referred to the pediatric department and special clinics of Isfahan Dental School in 2020 who were willing to participate in the study and complete the questionnaire, were recruited. The exclusion criteria included mothers who did not fill out the questionnaire completely, were illiterate, did not have the necessary cooperation to participate in the study, and were not in a physically and emotionally stable situation to complete the questionnaire.

Data sources/ measurementAfter completing the consent form of participation in the study, the respondents were asked to complete a questionnaire on anger expression. This questionnaire is part of Spielberg’s state-trait anger expression inventory (STAXI) [ 2 ]. Providing an overall measure of anger expression and control, this 57-item questionnaire includes six scales, five subscales, and an overall anger expression index. The anger expression (AX/EX) scale measures the expression and control of anger through the concept of "what my reaction or behavior is when I am usually furious or angry" [ 2 ].

### Variables

Anger expression and control are conceptualized in the form of four main components including (1) anger expression-out (AX-O), which includes expressing anger towards other people or objects present in the environment; (2) anger expression-in (AX-I), which indicates the orientation of anger within the person, and people who score high on this component may retain anger or suppress feelings of anger; (3) anger control-out (AC-O), which is defined as the control of feelings of anger by preventing the expression of anger towards other people or objects around; and (4) anger control-in (AC-I), which is related to controlling the suppression of emotions by calming down or being relaxed when getting angry [ 10 ]. 

Answer to the questions are rated on a four-point scale from "never = 1" to "always = 4". After reviewing and completing the questionnaires, the overall anger expression index, which provides an overall measure of anger expression based on individual scores of the scales mentioned, was calculated by the following equation: 

AX Index = (AX-O+AX-I) – (AC-O + AC-I) + 48

The fixed number 48 is included in the formula to eliminate the negative numbers. A person’s scores in this index can range from zero to 96 [ 10 ]. The content validity of the Persian version of this scale was reviewed and confirmed by experts in the study of Khodayari Fard *et al*. [ 10 ].

### Setting

The respondents were given 6-8 minutes to complete the anger expression questionnaire. Then, they were asked to watch the educational video on behavior management methods prepared by the professors of the pediatric department of Isfahan Dental School in 2018. To make this film, a 6-year-old child was trained as an actor after taking the consent of his parents, and behavior management techniques, including voice control, tell-show-do, pre-appointment behavior shaping, active restraint, passive restraint, mouthpiece, and general anesthesia were performed on him. All recorded videos were reviewed and evaluated by three pediatric dentists, and some behavior management techniques were re-filmed to demonstrate the technique accurately. The visual analog scale (VAS) was used to measure the mothers’ acceptance level after watching the video of each method.

This scale indicates a 100-mm long horizontal line with indicators at each end, ranging from "strongly agree" at the right end to "strongly disagree" at the left end. An individual places a cross (×) mark on the line to indicate their acceptance of each behavior management method. This value was measured with a ruler and used for the relevant analyses. Previous studies have shown that the results of the VAS scale are valid in assessing the parents’ acceptance of behavior management methods [ 11 - 12 ]. After watching each video of the behavior management method, the individual was given 10 seconds to determine their acceptance rate by placing a cross (×) mark on the horizontal line. 

Statistical methodsData analysis was performed by SPSS (Version 20.0) software using Spearman correlation coefficient and Mann-Whitney test.

## Results

A total of 110 mothers of children referred to the pediatric department and special clinics of Isfahan Dental School were included in this study. The mean age of the participating mothers was 38.06±5.70 years and that of their children was 7.35±2.72 years. Of mothers, 75.45% had a previous history of referral of one of their children to the dentist. The frequency distribution of mothers participating in the study in terms of education level, age, and gender of their children is presented in Table 1.

**Table 1:** Percentage frequency distribution of demographic information

Mother’s education	Primary education	High school diploma	Associate degree	Bachelor degree	Master’s degree	Ph.D.
N=110	4.55%	21.82%	10.91%	45.45%	14.55%	2.72%
	5	24	12	50	16	3
Child’s gender	Male	Female				
N=110	40.91%	59.09%				
	45	65				
Child’s age	1-6	6-12				
N=110	25.45%	74.55%				
	28	82				

Among the seven behavior management methods studied, the tell-show-do method, with a mean score of 7.46±2.76, was reported to have the highest acceptance rate and the active restraint method, with a mean score of 4.78±3.63, was reported to have the lowest acceptance rate. Further, there was no significant relationship between children's gender and mothers’ acceptance of behavior management methods (*p*> 0.05). The mean and standard deviation of mothers’ acceptance of behavior management methods are reported in Table 2. The mean total score of mothers' anger expression was 34.95 ±10.47.

**Table 2:** Mean and standard deviation of acceptance of behavior management methods

Behavior management method	Mean	SD
Pre-appointment behavior shaping	6.54	3.28
Tell-show-do	7.46	2.76
Voice control	6.13	2.98
Mouthpiece	6.19	3.26
Active restraint	5.31	3.32
Passive restraint	4.78	3.63
General anesthesia	5.35	3.78

To evaluate the mothers’ acceptance of behavior management methods and their anger expression, the Spearman correlation coefficient was used. The results showed only a significant inverse relationship between the mothers’ acceptance of the tell-show-do method and their anger expression level (*p*= 0.023). Additional data are available in Table 3. For a more precise assessment, the relationship between the acceptance of behavior management methods and anger expression subscales was examined separately. The results of the Spearman correlation coefficient (Tables 4 and 5) indicated no significant relationship between the acceptance of behavior management methods and the AC-I, AC-O and AX-O subscales separately (*p*> 0.05).

**Table 3:** Results of Spearman correlation coefficient for anger expression and acceptance of behavior management methods

	*p*	Spearman correlation coefficient
Anger expression-voice control	0.170	-0.132
Anger expression-tell-show-do	0.023	-0.215
Anger expression-pre-appointment behavior shaping	0.116	-0.151
Anger expression-active restraint	0.444	0.074
Anger expression-passive restraint	0.520	-0.062
Anger expression-mouthpiece	0.496	0.066
Anger expression-general anesthesia	0.484	0.067

**Table 4:** Spearman correlation coefficients of mothers’ anger expression-in and -out and their acceptance of behavior management methods

Behavior management method	Anger expression-in		Anger expression-out	
	*p*	Spearman correlation coefficient	*p*	Spearman correlation coefficient
Pre-appointment behavior shaping	0.318	0.096	0.283	-0.103
Tell-show-do	0.380	-0.084	0.162	0.134
Voice control	0.196	-0.124	0.839	-0.020
Mouthpiece	0.888	0.014	0.669	0.040
Active restraint	0.688	0.039	0.991	0.001
Passive restraint	0.821	0.022	0.662	0.042
General anesthesia	0.757	0.030	0.094	0.161

**Table 5:** Spearman correlation coefficients of mothers’ anger control-in and -out and their acceptance of behavior management methods

Behavior management method	Anger control-in		Anger control-out	
	*p*	Spearman correlation coefficient	*p*	Spearman correlation coefficient
Pre-appointment behavior shaping	0.434	0.075	0.083	0.166
Tell-show-do	0.156	0.136	0.097	0.159
Voice control	0.051	0.187	0.149	0.138
Mouthpiece	0.316	-0.096	0.742	-0.032
Active restraint	0.370	-0.086	0.199	-0.123
Passive restraint	0.183	0.128	0.570	0.055
General anesthesia	0.746	-0.031	0.153	-0.137

The assessment of other variables such as the relationship between age, mothers’ education, and previous experience of a pediatric dental visit and their acceptance of behavior management methods showed only a significantly positive relationship between the use of mouthpiece and mothers' age (*p*= 0.033). The analysis of the possible factors affecting the anger expression indicated no significant relationship between mothers’ ages and education and their anger expression level (*p*> 0.05). Further, there was no significant difference in the acceptance rate between mothers having daughters and those having sons (*p*> 0.05).

## Discussion

With an increase in parental involvement in decisions related to children’s behavior management methods used in pediatric dentistry, it is highly important to evaluate their acceptance of these methods and the factors affecting them. The present study was conducted to investigate the relationship between anger expression in mothers of children and their acceptance of behavior management methods.

According to the mean scores of acceptance of behavior management methods in Table 2, the tell-show-do method was more accepted by mothers than other methods, followed by the pre-appointment behavior shaping, using the mouthpiece, voice control, active restraint, and general anesthesia, respectively. The passive restraint method with a mean score of 4.78±3.63 was less accepted than other methods.

The tell-show-do technique is widely used to familiarize the patient with a new treatment process [ 13 ]. It includes an oral explanation of the treatment process through statements and words appropriate to the child's level of cognitive development, demonstrating the safety of the steps using visual, auditory, and tactile senses [ 14 ]. The pre-appointment behavior shaping technique is based on this psychological principle that the child imitates the behavior of others through observation. In this technique, an objective model such as another child of the same age receiving dental treatment or a video can be used [ 15 ]. Voice control is defined as speaking louder and changing the tone of voice in a controlled manner to direct the child’s behavior. The purpose of using this technique is to attract the child’s attention and cooperation and prevent avoidance and negative behaviors [ 15 ]. The use of protective restraints confines the child's freedom of movement, thereby reducing the risk of injury to the patient, staff, or parents during dental treatments. In the active restraint method, the dentist or dental assistant actively immobilizes the child by holding the head, arms, and body, and in the passive restraint method, the child is locked in a stationary device to control his/her movements [ 15 ]. The mouthpiece is a device that helps the cooperative child keep his mouth open. But in some cases, it is placed in the mouth when the child is uncooperative and refuses to open his/her mouth [ 3 ]. In some cases, the dentist has to perform dental treatment under general anesthesia [ 4 ].

Based on the studies conducted so far, the acceptance rate of a technique depends on many factors that have changed over time, such as the type of required treatment and its urgency, parents’ economic and cultural conditions, personality traits, culture and race, parenting styles and parents’ strictness, parents’ knowledge of behavior management methods, and explanation methods of these methods [ 6 , 16 - 21 ]. Moreover, a study showed parents’ satisfaction with the treatments received in medical emergencies could be influenced by their anger [ 22 ].

In the study by Jafarzadeh *et al*. [ 20 ] in 2014, the tell-show-do had the highest parents' attitude score between behavior management techniques used for treatment of children, and in the study by Hashemi *et al*. [ 23 ] in 2021; the tell-show-do method was more accepted by parents than other methods. However, the results of the two mentioned studies showed the lowest acceptance rate for the pharmaceutical methods, which was not consistent with the results of the present study and could be attributed to the novelty of the pharmaceutical methods and parents’ poor knowledge of these methods in the mentioned studies.

In the study of Eaton *et al*. [ 21 ] in 2005, the tell-show-do method was more accepted by parents, but pharmacological methods such as general anesthesia were more accepted than previous studies, and parents preferred these methods to other methods such as physical inhibition. In addition, the passive restraint method and placing the hand on the children’s mouth were less accepted than other methods, which was similar to the results of the present study. In a study conducted in Kuwait in 2013, the highest acceptance rate was reported for positive reinforcement and effective communication methods and the lowest acceptance rate was related to conscious relaxation techniques and placing the hand on the child's mouth [ 4 ].

The studies that have been done so far have examined different behavior management methods, and in each study, a number of these methods are in common with the present study. In general, it can be argued that the results of the present study are consistent with those of most recent studies and non-invasive methods such as tell-show-do are more acceptable, and physical inhibition methods such as passive restraint are less acceptable [ 8 , 24 - 26 ]. 

Diversity in the results of studies conducted in different countries at different times may be due to cultural and racial differences, changing attitudes and increasing parental knowledge over time, differences in the education method provided parents’ different experiences of dental treatments, and parents’ basic knowledge. Further, the presence of a control group in such studies can remarkably help to examine real differences between the samples.

There was a significantly negative relationship between mothers’ anger expression and their acceptance of behavior management methods only in the tell-show-do method (Table 3), and there was no significant relationship between the anger expression score and other methods, as mothers with lower anger expression scores were more likely to accept the tell-show-do method during their children’s dental treatment. Almost all national and international studies on parents’ acceptance of behavior management methods among different racial, social, cultural groups have shown that the tell-show-do method has the highest acceptance rate [ 2 , 24 - 25 , 27 - 31 ] and it seems that its superiority is not affected by other variables. Hence, it seems that anger affects the mothers’ acceptance of this method, and future studies are suggested to explore the effect of anger expression on the acceptance of other behavior management methods. 

As the first study to investigate the relationship between anger and acceptance of behavior management methods, this study indicated a relationship between anger expression and the acceptance of one of the behavior management methods (tell-show-do). Further studies in other communities with larger sample sizes and control groups are recommended to examine the other dimensions of anger using other anger expression assessment tools and employing other modalities to explain behavior management methods to parents to gain a better understanding of the relationship between anger and acceptance of behavioral management methods. The use of a live model and direct demonstration of using behavior management methods to parents may yield different results than those of the method used in this study and movie display. Mirmoeini *et al*. [ 32 ] in 2020 showed that the presentation and demonstration of behavior management methods affected the mothers' acceptance of these methods.

To perform a more precise evaluation, the relationship between the acceptance of behavior management methods and the anger expression subscales was examined separately. The results showed no significant relationship between the acceptance of behavior management methods and AX-O, AX-I, AC-O, and AC-I subscales separately. The analysis of other variables such as the relationship between mothers 'age and their acceptance of behavior management methods showed only a significantly positive association between the use of mouthpiece and mothers’ age, i.e. the higher the mothers’ age, the higher the acceptance of this method.

There was no significant relationship between mothers’ education level and their acceptance of behavior management methods. Several studies have also shown that parental education does not affect the acceptance of behavior management methods [ 4 , 21 , 27 , 33 - 34 ]. Some other studies have indicated no significant relationship between parents’ age and education level and their acceptance of behavior management methods [ 20 , 21 - 27 ]. 

There was no significant relationship between mothers’ previous experience of a pediatric dental visit and their acceptance of behavior management methods. Having previous experience of the pediatric dental visit and how the dentist manages the child's behavior may not affect the people’s perceptions of the educational videos provided, although the relationship of this factor with acceptance of behavior management methods has not been studied and needs further investigation.

There was no significant relationship between mothers’ age and education level and their anger expression level. Yousefi *et al*. [ 35 ] in 2015 and Asghari Moghadam *et al*. [ 36 ] in 2001 also reported that education level did not affect the anger expression rate.

Further studies are needed to investigate whether mothers' concern about the emotional problems and harm caused by using more aggressive behavior management techniques is affected by their tendency to express external and internal anger.

## Conclusion

There was a significant inverse relationship between acceptance of the tell-show-do behavioral management method and mothers’ anger expression. However, there is no significant relationship between other methods and mothers’ anger expression. According to this study, there was a relation between mothers' ages and their acceptance of using a mouthpiece.

## Acknowledgments

This work was supported by the Isfahan University of medical sciences.

## Conflict of Interest

The authors declare that they have no conflict of interest.
